# Causal associations between severe COVID-19 and diseases of seven organs: a proteome-wide mendelian randomization study

**DOI:** 10.3389/fgene.2024.1421824

**Published:** 2024-08-13

**Authors:** Yunhan Shen, Yi Zhang, Ye-yang Xu, Xinyi Li, Jiachen Wu, Hao Pei, Linyan Wang, Tiansheng Zhu

**Affiliations:** ^1^ College of Mathematics and Computer Science, Zhejiang A&F University, Hangzhou, China; ^2^ The First Affiliated Hospital of Zhejiang Chinese Medical University (Zhejiang Provincial Hospital of Chinese Medicine), Hangzhou, China; ^3^ Songyang County People’s Hospital, Lishui, Zhejiang, China; ^4^ MobiDrop (Zhejiang) Co., Ltd., Tongxiang, Zhejiang, China; ^5^ Zhejiang Provincial Key Laboratory of Ophthalmology, Eye Center, The Second Affiliated Hospital, School of Medicine, Zhejiang Provincial Clinical Research Center for Eye Diseases, Zhejiang Provincial Engineering Institute on Eye Diseases, Zhejiang University, Hangzhou, Zhejiang, China

**Keywords:** severe COVID-19, causal effect, mendelian randomization (MR), organ-related diseases, proteome

## Abstract

The coronavirus disease 2019 (COVID-19) pandemic poses an enormous threat to public health worldwide. Many retrospective studies and case reports to date have shown associations between severe COVID-19 and diseases of multi-organs. However, the research on the causal mechanisms behind this phenomenon is neither extensive nor comprehensive. We conducted a proteome-wide Mendelian randomization (MR) study using summary statistics from a Genome-Wide Association Studies (GWAS) of severe COVID-19 and diseases related to seven organs: lung, spleen, liver, heart, kidney, testis, and thyroid, based on the European ancestry. The primary analytical method used is the radial inverse variance-weighted (radial IVW) method, supplemented with the inverse variance-weighted (IVW), weighted-median (WM), MR-Egger methods. Our findings have confirmed the association between severe COVID-19 and multiple organ-related diseases, such as Hypothyroidism, strict autoimmune (HTCBSA), Thyroid disorders (TD), and Graves’ disease (GD). And we have also identified certain proteins that are associated with organ-related diseases, such as Superoxide Dismutase 2 (SOD2) and TEK Receptor Tyrosine Kinase (TEK), which are also considered potential drug targets. Phenotype scanning and sensitivity analyses were implemented to consolidate the results for Mendelian randomization. This study provides a compelling foundation for investigating COVID-19 caused diseases in future studies.

## Introduction

During the COVID-19 pandemic, the global public health system has faced a significant crisis. Infection with SARS-CoV-2 is associated with symptoms such as chest pain, difficulty breathing, and muscle pain (Ballering et al., 2022). According to a report from the World Health Organization on 9 August 2023, more than 760 million cases and 6.9 million deaths have been recorded globally since December 2019, but the actual numbers may be higher. Despite the effective control of COVID-19 effects post-vaccination, challenges persist (Hall et al., 2022).

Davis et al.'s review details the severe impact of long COVID-19 on organs such as the lungs, heart, pancreas, kidneys, spleen, and liver (Davis et al., 2023). Many patients present with multiple symptoms across multiple organ systems (Davis et al., 2021). Therefore, elucidating the correlation between severe COVID-19 (very severe respiratory confirmed COVID-19) and organ-related diseases holds significant value for public health.

Mendelian randomization (MR) is an analytical method for testing causal relationships between exposures or risk factors and clinically relevant outcomes (Davey Smith and Ebrahim, 2003). The impact of confounding variables and reverse causality on the precision of correlation discovery can be effectively reduced by Mendelian randomization. The number of publicly available Genome-wide association study statistics (GWAS) databases has increased in recent years, and Mendelian randomization is now widely applied in epidemiological research (Ge et al., 2023; Kim et al., 2023).

In previous studies, most Mendelian randomization studies on COVID-19 have focused more on causal analysis between single-type diseases (Li et al., 2022; Zhang et al., 2022). In this study, we systematically analyzed the association between severe COVID-19 and diseases related to seven organs, aiming to explore the causal relationship between severe COVID-19 and diseases in these seven organs. We used the significant single nucleotide polymorphisms (SNPs) in the severe COVID-19 phenotype as instrumental variables (IVs). However, due to the high false positive rate in MR analysis caused by the presence of numerous instrumental variables, and thanks to the irreplaceable role of proteomics research and the progress made in COVID-19 studies (Bi et al., 2022; Babacic et al., 2023), there are now COVID-19-related proteomics datasets available for effectively screening strongly associated instrumental variables. We utilized multi-organ proteomics data from COVID-19 autopsies to screen for corresponding organ diseases before conducting two-sample MR to explore this causal relationship (Nie et al., 2021). Through these analyses, we seek to gain deeper insights into the impact of severe COVID-19 on various organs, providing novel perspectives and treatment strategies for addressing COVID-19-related complications. Additionally, we utilized corresponding databases for drug target exploration and phenotype screening, and constructed Protein-Protein Interaction (PPI) networks.

## Materials and methods

### Overview

**FIGURE 1:**
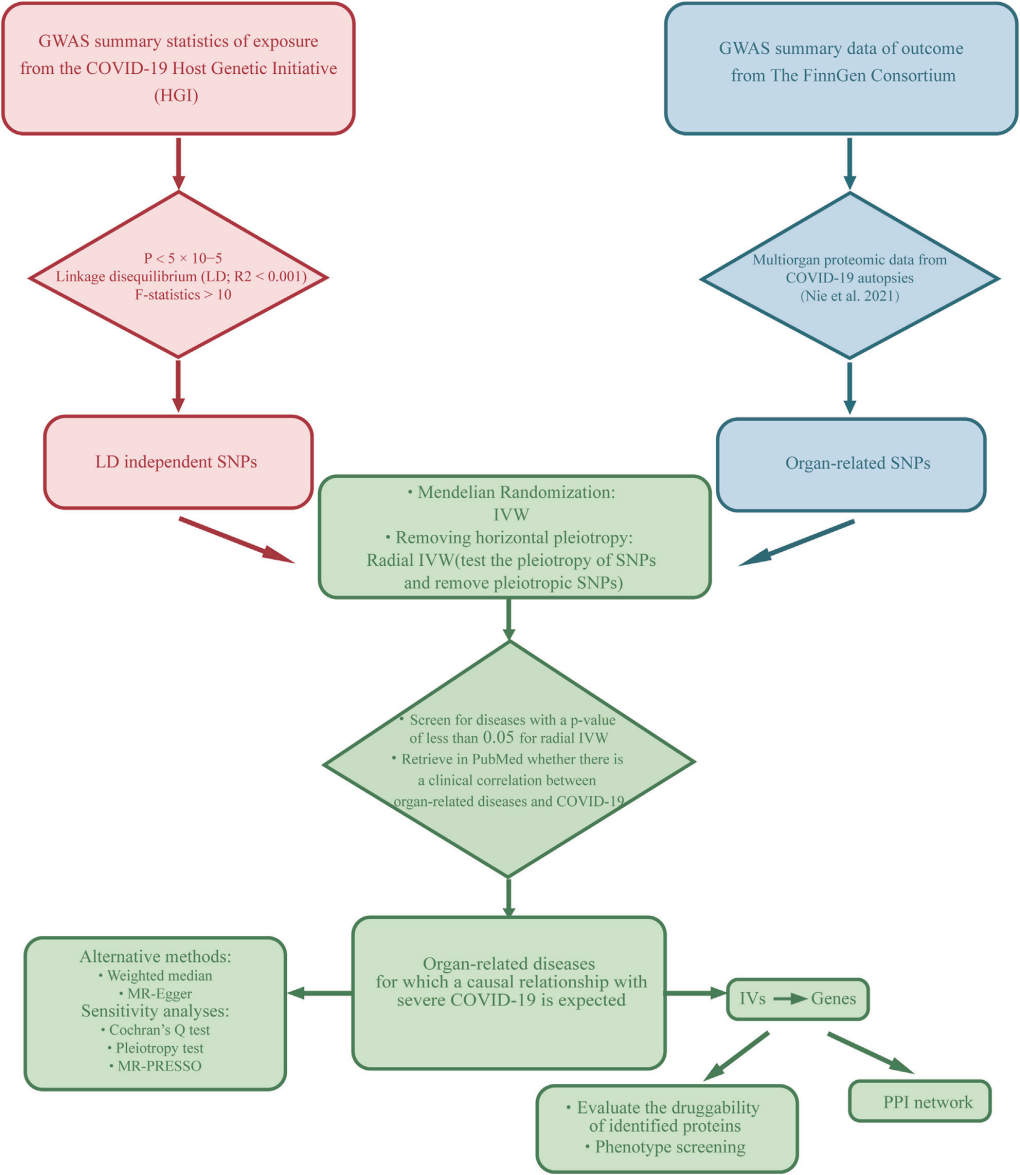
Description of the study design in this MR study. SNPs, single nucleotide polymorphisms; IVs, instrumental variables.

In this study, we used a two-sample MR analysis to explore the causal association between exposure and outcome and based on three hypotheses: first, there is a robust correlation between IVs and the exposure factor. Second, IVs are independent of confounding factors that are associated with the outcome. Third, IVs affect the outcome only through the exposure and not through any other mechanisms (Lawlor, 2016). We used the phenotype associated with severe COVID-19 as the exposure. Then, we selected diseases related to seven organs (lung, spleen, liver, heart, kidney, testis, thyroid) as the outcome. The specific workflow of the study is illustrated in Figure 1. In order to ensure the scientific validity and reliability of the MR analysis, we follow the STROBE-MR guidelines developed by Skrivankova (SAppendix 1) (Skrivankova et al., 2021).

### Data sources

We have used the GWAS summary statistics of severe COVID-19 phenotypes compiled from the COVID-19 Host Genetics Initiative (HGI). (Initiative et al., 2021) (https://www.covid19hg.org/results/r7/), which have been widely utilized in previous studies (Huang et al., 2022; Ge et al., 2023). The summary of the information for the corresponding data on severe COVID-19 is presented in Table 1. GWAS summary statistics of seven organ-related diseases were then filtered from The FinnGen Consortium (https://www.finngen.fi/en), and the information for specific diseases is presented in Supplementary Table 1. The proteomic data used to screen for organ-associated SNPs were obtained from Nie et al.’ study (Nie et al., 2021). In this study, using tandem mass tagging (TMT)-based shotgun proteomics, they quantified 11,394 proteins and found that 5,336 of them were significantly dysregulated in at least one organ in COVID-19 patients (Lung:1606; Spleen:1726; Liver: 1969; Heart: 919; Kidney: 2227; Testis: 10; Thyroid: 1297) (Nie et al., 2021). In our study, we directly downloaded the list of differential proteins provided by this research for subsequent analysis.

**TABLE 1:** Description of the data involved in Mendelian randomization studies.

Exposure	Total cases	Total controls	Number of SNPs	Ancestry
COVID‐19 (severe vs population)	13769	1072442	12174527	European
Outcome				
Pneumoniae	63377	348804	21306349	European
*Streptococcus* pneumoniae	1339	344010	21304820	European
Liver diseases	11934	400247	21306349	European
Kidney cyst	1874	408319	21306310	European
Other specified disorders of kidney and ureter	948	408319	21306293	European
Other disorders of kidney and ureter	3862	408319	21306349	European
Hypothyroidism, strict autoimmune	45321	298847	21304850	European
Thyroid disorders	62464	349717	21306349	European
Graves’ disease	3176	409005	21306349	European

SNPs, single nucleotide polymorphisms.

### IV selections

We selected appropriate IVs for MR analysis from two different GWAS summary statistics through a rigorous series of screens to meet the three fundamental assumptions. Initially, a genome-wide significance threshold (*p* < 5 × 10^−5^) to select SNPs that are stably associated with severe COVID-19, and the reasonableness of this threshold setting has been confirmed in previous studies (Ge et al., 2023).

The characteristic of Linkage Disequilibrium (LD) is the non-random correlation between two or more genetic loci with advantageous hereditary traits. r^2^ > 0.001, kb > 10000 was set to screen the appropriate SNPs, thus reducing the impact of chain-imbalanced LD on the results. The threshold used in this study was determined by analyzing a large dataset including European genomes. Previous multiorgan proteomics data on COVID-19 autopsies were used to screen for SNPs for corresponding organ diseases that correlate with COVID-19 (Nie et al., 2021). To ensure a strong association between exposure and outcome, we estimated the F‐statistics to assess the strength of IVs, which is calculated by dividing the square of beta by the square of the standard error (SE). Strong IVs with F-statistics greater than 10 were used (Burgess et al., 2011). Furthermore, we conducted phenotype screening of IVs to confirm the exclusion of confounding factors affecting the results.

### Statistical analysis

In this MR study, we used radial inverse variance-weighted (radial IVW) and inverse variance-weighted (IVW) as the primary analysis method. The Radial MR method removes the corresponding outliers and reduces the effect of pleiotropy on the results (Bowden et al., 2018). IVW assumes that all genetic variants can be used as valid instrumental variables. it is very stable in detecting causality and can provide impact estimates with significantly lower variance. The *p*-value of radial IVW less than 0.05 was then used as a screening criterion to screen out relevant diseases for the next analysis. Building upon the second-order weighted radial framework, the study also supplemented two additional methods, MR-Egger regression and weighted median (WM). The MR-Egger method was able to analyze the possible genetic pleiotropy of the IVs involved in the study (Bowden et al., 2015). In MR-Egger regression, if the intercept term is zero or lacks statistical significance, the regression slope can be interpreted as the estimated causal effect of exposure on the outcome. The weighted median (WM) method can provide consistent causal estimates in the presence of numerical errors for half of the IVs (Bowden et al., 2016). According to the standard of previous studies (Freuer and Meisinger, 2023; Wang et al., 2023), it is generally believed that in a study, the radial IVW result less than 0.05 indicates statistical significance and suggests the presence of a corresponding causal relationship.

Then we evaluate the heterogeneity of IVs based on the second-order weighted radial using I^2^ statistics and Cochran’s Q test. When the I^2^ statistic fell between 0% and 25%, it indicated only mild heterogeneity. When the value falls within the range of 25%–50%, it indicates moderate heterogeneity, and an I^2^ statistic above 50% indicates the presence of significant heterogeneity. If the *p*-value of Cochran’s Q test is less than 0.05, it indicates the presence of heterogeneity. Conversely, if the *p*-value is equal to or greater than 0.05, it indicates the absence of heterogeneity. Additionally, the pleiotropy test and Mendelian randomization pleiotropy residual sum and outlier (MR-PRESSO) test are employed to assess horizontal pleiotropy. If the *p*-value of the pleiotropy test is less than 0.05, it indicates the presence of pleiotropy; if the *p*-value is greater than 0.05, it suggests no pleiotropy. In the MR-PRESSO test (Verbanck et al., 2018), a global test *p*-value less than 0.05 indicates the presence of pleiotropy, while a *p*-value greater than 0.05 suggests no pleiotropy. All these analyses were conducted using the “TwoSampleMR”, “RadialMR”, “MendelianRandomization”, and “MR-PRESSO” packages in R (version 4.2.2).

### Druggable proteins identification, phenotype scanning and PPI network

We mapped the SNPs obtained from the MR analysis to their corresponding proteins, and assessed the druggability of these proteins. We searched these proteins in the DrugBank database. For the proteins identified in the drug database, we documented information regarding the drug names and the associated diseases.

We also conducted phenotype scanning, searching previous GWAS to reveal the associations of identified proteins with other traits. Phenotype scanning was performed using the LDtrait Tools (https://ldlink.nih.gov/). SNPs were considered pleiotropic under the following criteria (Ballering et al., 2022): significant associations at the genome-wide level (*p* < 5 × 10^−5^) (Hall et al., 2022); GWAS conducted in European ancestral populations (Davis et al., 2023); SNPs associated with known risk factors, including metabolic traits, proteins, or clinical characteristics.

To further evaluate the protein networks of individual organ proteins associated with COVID-19, we constructed protein interaction networks using the STRING (Search Tool for the Retrieval of Interaction Gene/Proteins) database (https://cn.string-db.org/cgi/input.pl).

## Results

**FIGURE 2:**
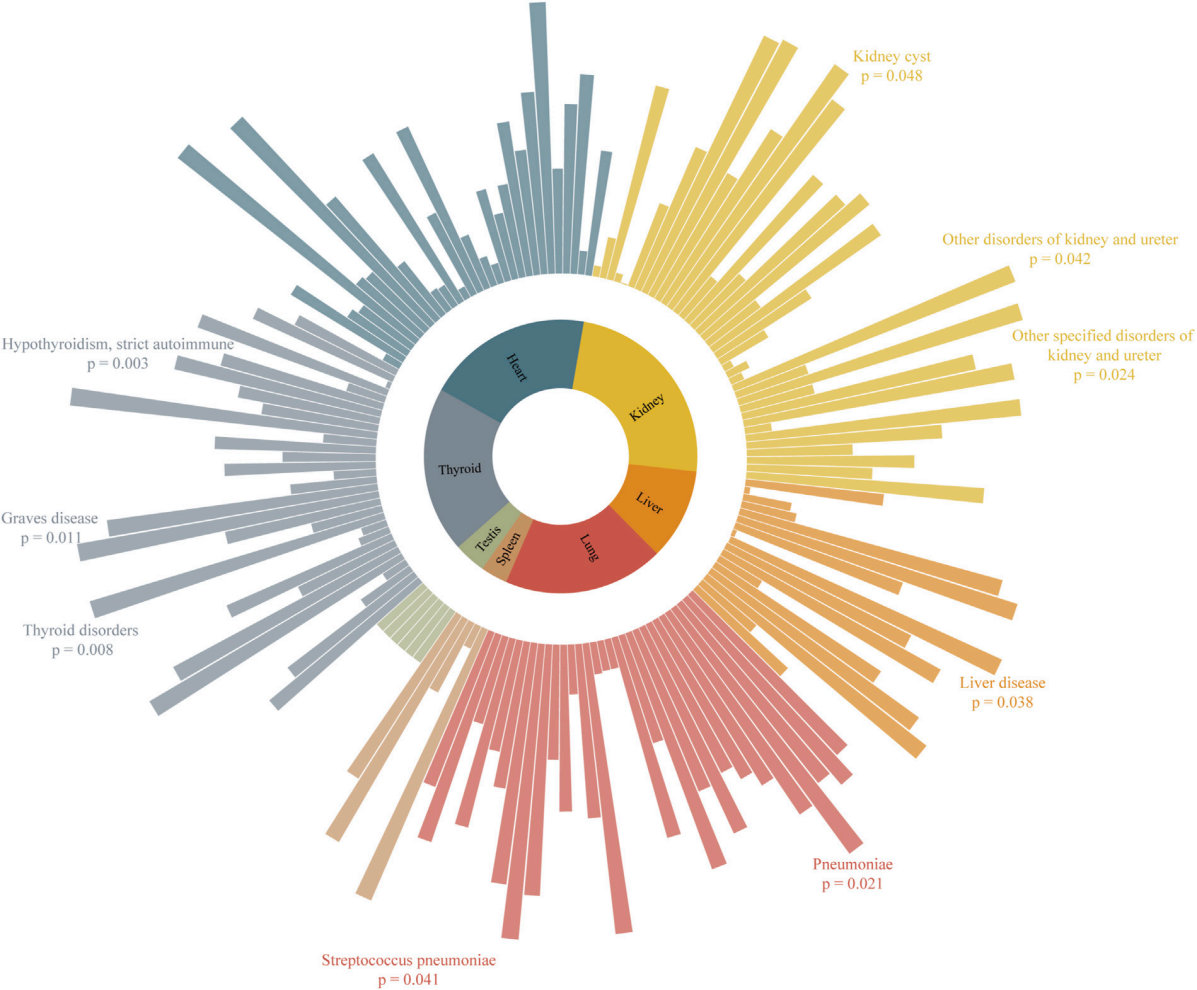
Association of severe COVID-19 in MR analysis with organ-related diseases. Higher bars represent lower *p*-values and stronger causal associations.

The results of the MR analysis for all diseases are presented in Figure 2 and Supplementary Table 1, showing both IVW and radial IVW results. We selected 13 diseases with positive radial MR results and conducted a literature search on PubMed. This confirmed that there is clinical evidence for nine of these diseases—namely, pneumonia, *Streptococcus* pneumoniae (SP), liver disease, kidney cyst (KC), other specified disorders of the kidney and ureter, other disorders of the kidney and ureter, thyroid disorders (TD), Graves’ disease (GD), and hypothyroidism (HTCBSA). Detailed information on these diseases can be found in Table 1. All SNPs used for the analysis are listed in Supplementary Table 2.

### The casual effect of severe COVID‐19 on lung-related diseases

**FIGURE 3:**
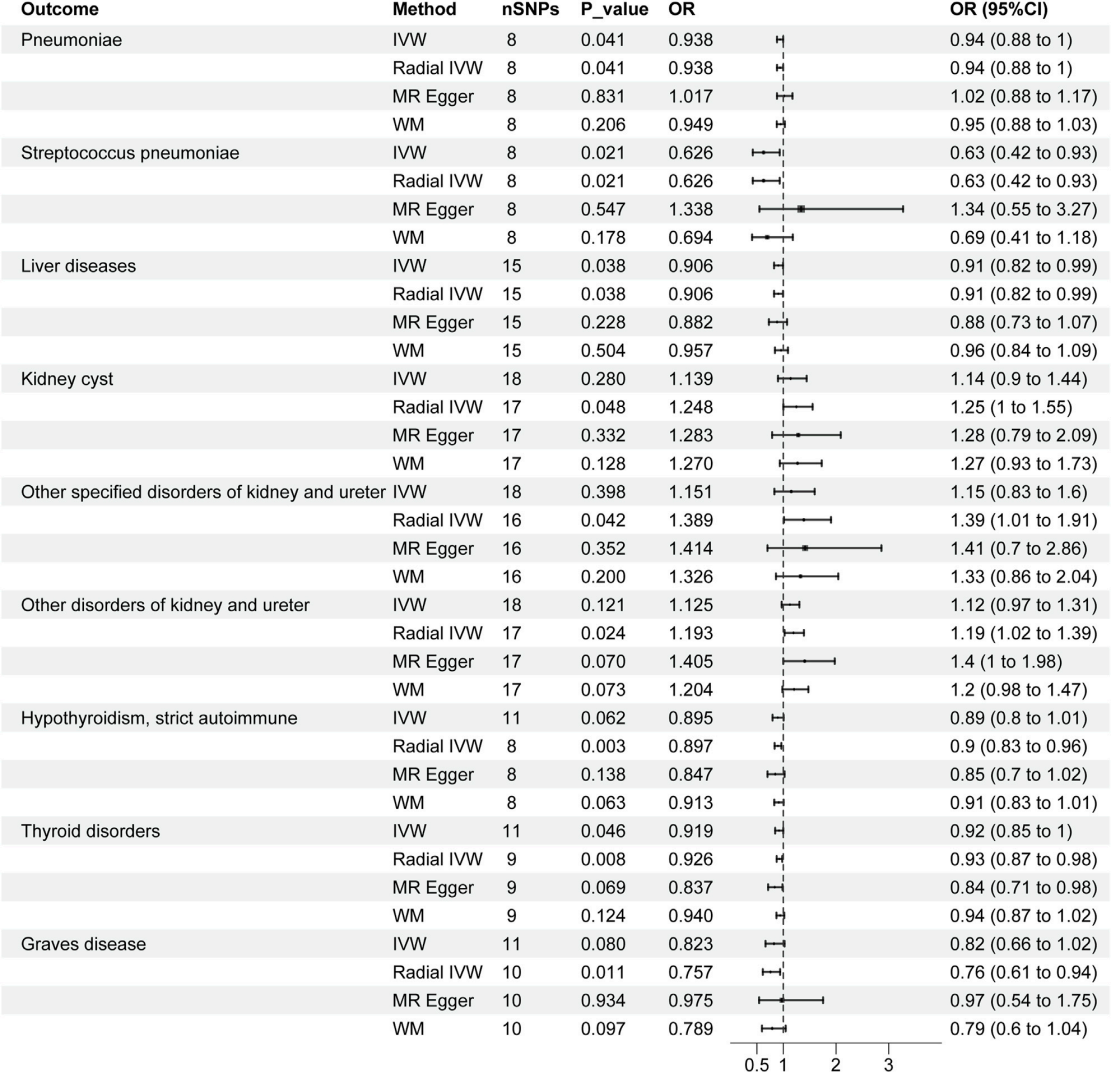
Forest plots of causal effects of severe COVID‐19 in organ-related disease. CI, confidence interval; OR: odds ratio; SNPs, single nucleotide polymorphisms.

According to Figure 3, the radial IVW analysis indicates a causal association between severe COVID-19 and pneumoniae (OR and 95% CI: 0.938, 0.88 to 1.00; *p* = 0.041). Due to the absence of outliers identified through second-order weighted radial regression, the results of the original IVW and radial IVW remain consistent. The results from MR-Egger (OR and 95% CI: 1.017, 0.88 to 1.17; *p* = 0.831) and WM (OR and 95% CI: 0.949, 0.88 to 1.03; *p* = 0.206) did not show significant associations. According to the Cochran’s Q test results (I^2^ = 0.0%, *p* = 0.754) in Table 2, there was no heterogeneity observed among severe COVID-19 and pneumoniae. In addition, the pleiotropy test (*p* = 0.270) and MR-PRESSO results (*p* = 0.791) indicate no horizontal pleiotropy between severe COVID-19 and pneumonia.

**TABLE 2:** Heterogeneity and pleiotropy tests of the Mendelian randomization studies.

Outcome	Cochran’s Q test		Horizontal pleiotropy		MR‐PRESSO	
	I^2^	*p*-Value	Egger intercept	*p*-Value	*p*-Value	Outliers
Pneumoniae	0.0	0.754	−0.009	0.270	0.791	0
*Streptococcus* pneumoniae	4.2%	0.381	−0.081	0.115	0.428	0
Liver diseases	0.0	0.707	0.003	0.769	0.715	0
Kidney cyst	0.0	0.724	−0.003	0.903	0.762	0
Other specified disorders of kidney and ureter	0.0	0.878	−0.002	0.956	0.890	0
Other disorders of kidney and ureter	0.0	0.914	−0.020	0.309	0.916	0
Hypothyroidism, strict autoimmune	0.0	0.843	0.007	0.551	0.848	0
Thyroid disorders	0.0	0.605	0.012	0.236	0.620	0
Graves’ disease	0.0	0.803	−0.028	0.392	0.793	0

In Figure 3, the IVW results for severe COVID-19 and SP were consistent with the radial IVW results, both of which were significant (OR and 95% CI: 0.626, 0.42 to 0.93; *p* = 0.021). MR-Egger (OR and 95% CI: 1.388, 0.55 to 3.27; *p* = 0.547) and WM (OR and 95% CI: 0.694, 0.41 to 1.18; *p* = 0.178) analyses do not yield significant results. The results of Cochran’s Q test (I^2^ = 4.2%, *p* = 0.381) in Table 2 indicate no heterogeneity between various COVID-19 phenotypes and SP. Pleiotropy test (*p* = 0.115) and MR‐PRESSO results (*p* = 0.428) also fail to demonstrate pleiotropy between the two.

### The casual effect of severe COVID‐19 on liver-related diseases

According to the radial IVW results in Figure 1A, there was a causal association between severe COVID-19 and liver diseases (OR and 95% CI: 0.906,0.82 to 0.99; *p* = 0.038). A correlation was also not presented in the other models. According to the results of Cochran’s Q test (I^2^ = 0.0%, *p* = 0.707) in Table 2, no heterogeneity was found between the results of COVID-19 and liver diseases, and the results of the pleiotropy test (*p* = 0.769) and MR-PRESSO (*p* = 0.715) similarly showed no pleiotropy.

### The casual effect of severe COVID‐19 on kidney-related diseases

According to Figure 3, after employing radial MR to remove horizontal pleiotropy from IVs, the VW analysis of severe COVID-19 and KCI shows an association (OR and 95% CI: 1.248,1.00 to 1.55; *p* = 0.048), and the global IVW results did not show a correlation (OR and 95% CI: 1.139,0.90 to 1.44; *p* = 0.280). No significant associations were found in the other models. According to Table2, Cochran’s Q test (*p* = 0.724), pleiotropy test (*p* = 0.903) and MR-PRESSO (*p* = 0.762) also showed no interference from heterogeneity and horizontal pleiotropy.

In both types of other disorders of kidney and ureter, the overall IVW did not show a significant association, but after removing outliers, the radial IVW analyses for both exhibited significant results (Figure 3). Furthermore, results from Cochran’s Q test, pleiotropy test, and MR‐PRESSO all indicate the absence of heterogeneity and horizontal pleiotropy interference (Table 2).

### The casual effect of severe COVID‐19 on thyroid-related diseases

In the IVW analyses, a corresponding causal relationship between severe COVID-19 and TD is found (OR and 95%CI: 0.919, 0.85 to 1.00; *p* = 0.046) (Figure 3). After conducting second-order weighted radial regression, it was found that the presence of two outliers could cause horizontal pleiotropy in the MR analysis. Despite the removal of horizontal pleiotropy, the radial IVW results remain significant (OR and 95%CI: 0.926, 0.87 to 0.98; *p* = 0.008) (Figure 3). The Cochran’s Q test results indicate no heterogeneity between severe COVID-19 and TD. Furthermore, both pleiotropy test and MR-PRESSO results suggest that the use of radial MR, after removing outliers, has mitigated the interference of horizontal pleiotropy (Table 2).

The results of Severe COVID-19 and HTCBSA did not present a correlation under the IVW model (OR and 95%CI: 0.895, 0.80 to 1.01; *p* = 0.062). Following the exclusion of three outliers’ impact on horizontal pleiotropy, the results under the radial IVW model showed significance (OR and 95%CI: 0.897, 0.83 to 0.96; *p* = 0.003) (Figure 3). Moreover, Cochran’s Q test results (I^2^ = 0.0%, *p* = 0.843) indicated the absence of heterogeneity afterward, and pleiotropy test (*p* = 0.551) and MR‐PRESSO (*p* = 0.848) results also demonstrated the complete removal of horizontal pleiotropy (Table 2).

The IVW analysis of severe COVID-19 and GD risk as shown in Figure 3 also did not show an identifiable causal relationship (OR and 95%CI: 0.823, 0.66 to 1.02; *p* = 0.080). After removing the influence of a single outlier on the outcomes, the radial IVW results suggest the existence of a correlated causal relationship (OR and 95%CI: 0.757, 0.65 to 0.94; *p* = 0.011). The results of MR-Egger and WM did not present a recognizable causal relationship. Results from Cochran’s Q test (I^2^ = 0.0%, *p* = 0.803), pleiotropy test (*p* = 0.392), and MR‐PRESSO (*p* = 0.793) also indicate no interference from heterogeneity and horizontal pleiotropy (Table 2).

### Druggability of identified proteins, phenotype scanning and PPI network

Among them, Superoxide Dismutase 2 (SOD2, P04179) and TEK Receptor Tyrosine Kinase (TEK, Q02763) have been confirmed as drug targets for COVID-19 (Supplementary Table 3). Desmoplakin (DSP, P15924) is a relevant drug target for the treatment of pneumonia, while SOD2 is used in the treatment of Hepatitis B. Currently, no drug targets have been found for the treatment of kidney and thyroid-related diseases. Information on drug targets and their associated drugs and diseases can be found in Supplementary Table 3.

Through phenotype scanning, we did not find any pleiotropic SNPs that could interfere with the experimental results. DSP is associated with idiopathic pulmonary fibrosis, lung dysfunction, chronic obstructive pulmonary disease, interstitial lung disease, and advanced glycation end product receptor levels. Phospholipid Scramblase 1 (PLSCR1, O15162) is associated with COVID-19. Lamin A/C (LMNA, P02545) is correlated with white blood cell count, and Nephronectin (NPNT, Q6UXI9) has been identified as a major gene influencing lung function, respiratory system diseases, chronic obstructive pulmonary disease, and peak expiratory flow rate.

After uploading all the genes used as instrumental variables in MR analysis to the STRING online database, we obtained a PPI network with color modification (Supplementary Figure 1).

## Discussion

As shown in previous studies, long and severe COVID-19 affect multiple organ systems (Davis et al., 2023). We further verified the association between severe COVID-19 and some organ-related diseases, such as Hypothyroidism, strict autoimmune (HTCBSA), Thyroid disorders (TD), and Graves’ disease (GD). This study builds upon the research of Nie et al. (Nie et al., 2021), further exploring the existing associations between COVID-19 and potential organ diseases, providing valid support for previous clinical results and retrospective studies.

Our study demonstrated an observable causal relationship between severe COVID-19 and pneumonia, as well as between severe COVID-19 and *streptococcus* pneumoniae (SP) (*p* = 0.021 and *p* = 0.041). The damage of COVID-19 to lung has long been confirmed by numerous studies (Berlin et al., 2020; Blanco et al., 2021; Su et al., 2021). Pneumonia is one of the main clinical manifestations of severe forms of COVID-19 (Li et al., 2020). The study by Angela et al. also confirmed the existence of an effect of COVID-19 on SP (Brueggemann et al., 2021) O'Toole’s study revealed the multifaceted impact of COVID-19 on bacterial infections, with the presence of infectious bacteria in patients admitted to the hospital with severe COVID-19 (O’Toole, 2021). It is worth noting that our study did not observe a potential causal relationship between idiopathic pulmonary fibrosis or interstitial lung disease and COVID-19 (*p* = 0.329 and *p* = 0.436). However, Wendisch et al.'s study established a correlation between COVID-19 and idiopathic pulmonary fibrosis (Wendisch et al., 2021). Additionally, several previous studies have confirmed that COVID-19 can lead to interstitial lung disease (Myall et al., 2021; Barash and Ramalingam, 2023).

In our MR analysis, we observed a causal relationship between severe COVID-19 and liver disease (*p* = 0.0384). In the review by Dufour et al., they are pointed out that liver injury may be caused by multiple factors, including the direct cytopathic effects of viruses, exaggerated systemic immune responses, vascular damage, coagulation disorders, and drug use (Dufour et al., 2022). In our study, although no significant causal association between severe COVID-19 and chronic hepatitis was found (*p* = 0.053), Dufour et al. noted that the risk of adverse outcomes after SARS-CoV-2 infection was increased in patients with chronic liver disease and cirrhosis (Dufour et al., 2022). Based on this finding, we reasonably speculate that there may be a clinical association between severe COVID-19 and chronic hepatitis. It is important to note that MR analysis relies on statistical models and data analysis, which may not fully capture the complexities of the real world. Due to the possibility of false negatives in genetic marker screening and validation during the MR analysis process, some causal relationships may not have been fully captured. According to Portincasa et al.‘s study, patients with COVID-19 are usually associated with metabolic disease, and in the presence of metabolic abnormalities, COVID-19 may exacerbate nonalcoholic fatty liver disease through the interaction of inflammatory pathways and direct or indirect effects of the virus on the liver (Portincasa et al., 2020). Our study still fails to reflect this point (*p* = 0.841).

In studies of severe COVID-19 and kidney-related diseases, COVID-19 is thought to trigger chronic kidney diseases and acute kidney injury (Pecly et al., 2021; Long et al., 2022). in our study, although an association between severe COVID-19 and chronic kidney diseases was not found, the presence of a false negative (*p* = 0.083) could not be ruled out. In addition, we also found an association between severe COVID-19 and kidney cyst (*p* = 0.048), although we did not find direct evidence of an association, as chronic kidney diseases may trigger kidney cyst. In the study by Chan et al., it was found that out of 3993 COVID-19 hospitalized patients, 1835 (46%) patients developed acute kidney injury (Chan et al., 2021). However, in this MR analysis, none of the individual acute kidney diseases demonstrated an association with COVID-19. (Supplementary Table 1).

The thyroid disorders caused by COVID-19 has been widely demonstrated (Khoo et al., 2021; Lui et al., 2024). HTCBSA is one of the major subtypes of hypothyroidism. Some previous studies have provided evidence of COVID-19-related hypothyroidism (Muller et al., 2020; van Gerwen et al., 2020; Burekovic et al., 2022). In our study, a causal association between severe COVID-19 and HTCBSA was observed, but results for some other rare subtypes of hypothyroidism were negative. In a previous MR analysis, COVID-19 was confirmed to be a risk factor for hypothyroidism and there was no evidence to support an association between COVID-19 and hyperthyroidism (Zhang et al., 2022). This is consistent with the results we obtained. It is important to note that while our study did not find direct evidence linking severe COVID-19 with hyperthyroidism, we did observe a correlation between severe COVID-19 and GD (*p* = 0.011). COVID-19 has been recognized as a potential trigger for GD, and there have been several case reports and studies of patients who developed symptoms of GD after infection with COVID-19 (Harris and Al, 2021; Tutal et al., 2022). For such results above, we believe that although GD is a common cause of hyperthyroidism, there are still other causes that induce hyperthyroidism, and severe COVID-19 and hyperthyroidism may not be directly linked.

In our MR analysis of severe COVID-19 and heart-related diseases, only one condition (cardiovascular disorders originating in the perinatal period, *p* = 0.024) showed a correlation. However, there is a lack of corresponding clinical studies to validate the potential causal relationship with COVID-19. Although some studies suggest that COVID-19 infection may increase the risk of myocarditis and pericarditis (Lindner et al., 2020; Patone et al., 2021), our research did not find a direct causal relationship between the two.

In our research, we only identified one spleen-related disease associated with COVID-19 (spleen cyst, *p* = 0.047). Despite studies by Jana et al. indicating that severe COVID-19 pneumonia can lead to loss of B cells in the bone marrow or spleen (Ihlow et al., 2021), there is no direct evidence to establish a direct or indirect association between COVID-19 and spleen cyst.

A large number of previous studies have shown that COVID-19 affects the human reproductive system (La Marca et al., 2020; Seymen, 2020). In fatal cases of COVID-19 infection, viral infection associates with activation of interferon pathways and downregulation of testis-specific genes involved in spermatogenesis (Basolo et al., 2023). However, due to the design of our methods, after multiple layers of screening, we were unable to find IVs that could be used in this study to infer the potential association that exists between sever COVID-19 and testis. This limitation highlights the challenges we face in studying the effects of COVID-19 on the male reproductive system and suggests directions for future research, such as improving data collection and research methods to better understand the specific mechanisms by which the virus affects the testis.

### Limitations

While this MR study offers a comprehensive and effective causal analysis for COVID-19 and various organ-related diseases, there are still some limitations. First, COVID-19 presents with a wide range of clinical manifestations and comorbidities, and some of these potential factors may predispose to the development of related diseases. In this study, although we used proteomic data related to COVID-19 for screening to minimize confounding, we could not guarantee that all potential factors were excluded. Second, in this study, we only used Genome-Wide Association Studies (GWAS) summary data from European populations due to the lack of data from other populations, and therefore we cannot guarantee that our conclusions will hold true in other populations. Finally, although we ruled out weak IVs, pleiotropy, and LD through our experimental design, the IVs we used usually failed to account for most of the variance, which may result in MR analyses that do not have a high degree of statistical validity and are at risk of false-negative or false-positive results.

## Conclusion

Our research has confirmed the association between severe COVID-19 and multiple organ-related diseases. However, with some proven organ-related diseases, such as chronic hepatitis, chronic kidney disease and hyperthyroidism, we did not find a causal link. In addition, we have identified some proteins associated with organ-related diseases and corresponding drug targets. We hope that future MR studies can utilize larger and more precise GWAS data to refine our findings.

## Data Availability

Publicly available datasets were analyzed in this study. This data can be found here: The COVID-19 Host Genetics Initiative (HGI) data repository (https://www.covid19hg.org/results/r7/) and the FinnGen database (https://www.finngen.fi/en).
