# Supplementary material for: Causal associations between severe COVID-19 and diseases of seven organs: a proteome-wide mendelian randomization study
Source: Front Genet. 2024 Aug 13;15:1421824. doi: 10.3389/fgene.2024.1421824 (PMC11347274; doi:10.3389/fgene.2024.1421824)

SFigure 1. PPI network of individual organ proteins associated with COVID-19.

A. String network analysis of lung

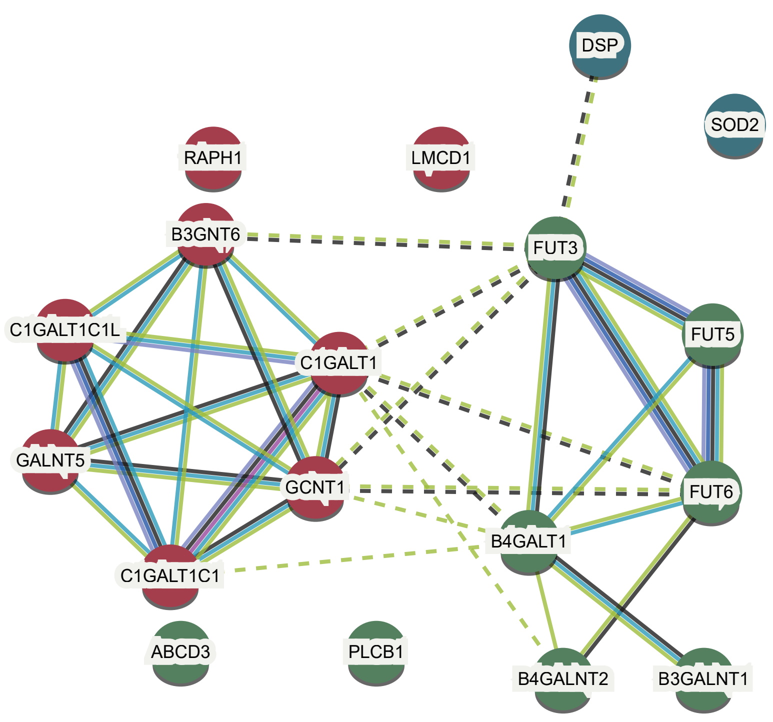

B. String network analysis of liver

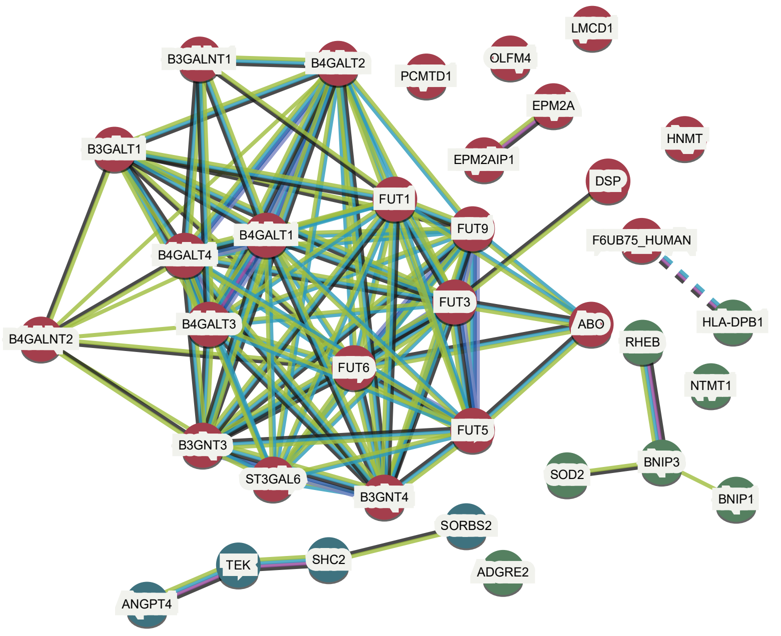

C. String network analysis of kidney

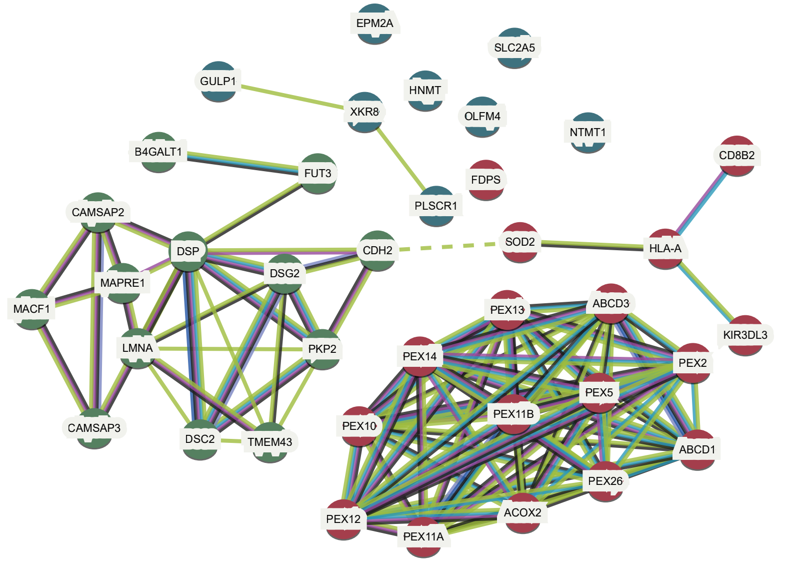

D. String network analysis of thyroid

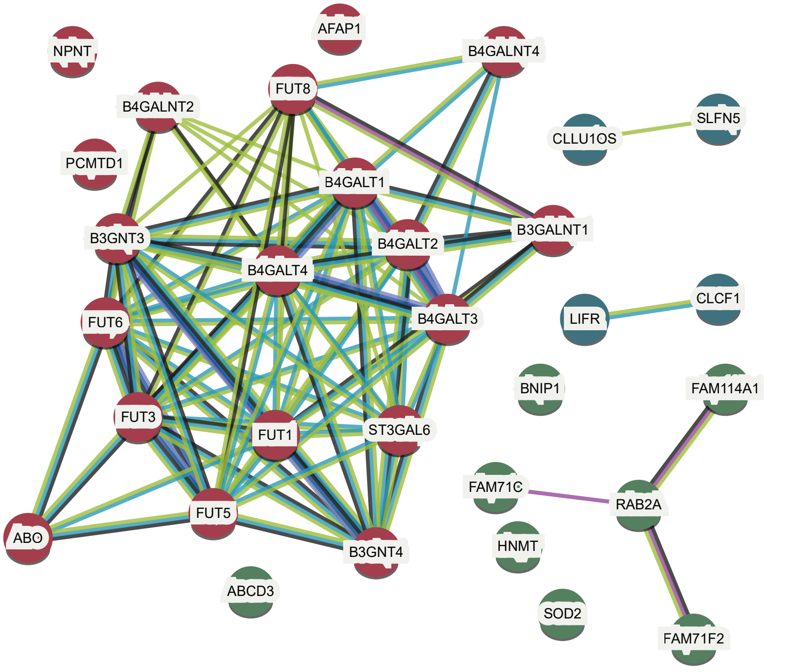

Supplement: Supplementary file 1 [file DataSheet2.PDF]
